# Comparative Mitogenomic Analyses of *Psectrocladius* (Diptera: Chironomidae)

**DOI:** 10.3390/insects16040420

**Published:** 2025-04-16

**Authors:** Xue-Yao Chen, Xiu-Ru Xiao, Yan Zhang, Zhi-Chao Zhang, Dong-Sheng Zhang, Zheng Liu, Xiao-Long Lin

**Affiliations:** 1 Engineering Research Center of Environmental DNA and Ecological Water Health Assessment, Shanghai Ocean University, Shanghai 201306, China; xychen23333@proton.me (X.-Y.C.); zyxr1023@hotmail.com (X.-R.X.); 17856074939@163.com (Y.Z.); zzc514644@gmail.com (Z.-C.Z.); dszhang@shou.edu.cn (D.-S.Z.); 2 Shanghai Universities Key Laboratory of Marine Animal Taxonomy and Evolution, Shanghai Ocean University, Shanghai 201306, China; 3 Department of Stratigraphy and Paleontology, Geological Museum of China, Beijing 100034, China; 4 Laboratory of Geo-Specimens Study and Testing, Geological Museum of China, Beijing 100034, China

**Keywords:** characteristics, Orthocladiinae, mitogenome, phylogeny

## Abstract

*Psectrocladius*, a genus within the species-rich subfamily Orthocladiinae (Diptera: Chironomidae), remains poorly resolved in molecular phylogenetics due to limited available molecular data. Here, we sequenced and analyzed the complete mitogenomes of five *Psectrocladius* species, using two *Rheocricotopus* species as outgroups. Our results reveal that the mitogenomes of *Psectrocladius* are structurally conserved and retain a presumed ancestral gene order. The nucleotide composition of these newly generated mitogenomes exhibits a pronounced A + T bias, which is characteristic of typical insect mitogenomes. The substitution rates, estimated using Ka/Ks ratios, indicate that all protein-coding genes are under purifying selection. The strongest purifying selection pressure was observed in the *CO1* gene, while the weakest was in the *ND5* gene. Both the maximum likelihood and Bayesian inference trees consistently show the following topology: ((((*P. schlienzi* + *P. bisetus*) + *P. barbimanus*) + *P. oligosetus*) + *P. aquatronus*). This study provides key insights into chironomid mitogenomes and their gene properties, offering valuable reference data for future research.

## Simple Summary

Mitochondrial genomes (mitogenomes) serve as critical molecular tools for advancing taxonomic and phylogenetic studies in insects. Despite the widespread application of mitogenomes as robust molecular markers in Diptera systematics, mitogenomic resources for *Psectrocladius* are exceptionally scarce, and the phylogenetic relationships within this genus have yet to be conclusively determined. In this study, the whole mitogenome sequences of five *Psectrocladius* species are reported for the first time. Our study offers valuable reference data on chironomid mitogenomes and provides critical insights into the properties of their constituent genes.

## 1. Introduction

Mitochondrial genomes (mitogenomes) are important molecular markers widely used for studies on phylogeny, evolutionary history, speciation, and phylogeography in insects owing to their maternal inheritance and high substitution rates [1,2]. Insect mitogenomes, characterized by distinctive genetic features, typically range in size from 14,000 to 20,000 base pairs (bp) in insects. These circular, double-stranded molecular structures encode a conserved set of 37 genes, which include 13 protein-coding genes (PCGs), two ribosomal RNA (rRNA) genes, 22 transfer RNA (tRNA) genes, and one non-coding control region (CR), which is involved in replication and transcription regulation [3,4]. The structure of insect mitogenomes is generally conserved, with gene arrangements similar to those of the ancestral insects, providing valuable insights into morphological classification and evolutionary relationships [5]. With the advancement of next-generation sequencing technologies, the number of complete insect mitogenomes has increased substantially, facilitating comparative studies of mitochondrial structure and evolutionary history at various taxonomic levels [6,7]. Due to their small genome size, maternal inheritance, low recombination rates, and rapid evolution rate, mitogenomes are considered powerful tools for phylogenetic and evolutionary analyses across diverse insect taxa [8,9].

Chironomidae (Diptera) is one of the most species-rich and widely distributed insect families. This family has undergone extensive adaptive radiation, enabling it to occupy a broader range of microhabitats than any other aquatic insect group, making it a crucial component of both terrestrial and aquatic ecosystems [10,11,12]. The genus *Psectrocladius,* which belongs to Orthocladiinae, is one of the most abundant and ecologically diverse groups within Chironomidae [13]. Due to its high habitat diversity, Orthocladiinae is recognized as one of the most taxonomically complex subfamilies in Chironomidae [14]. The larvae of *Psectrocladius* are distributed across all biogeographic regions except the Antarctic and Australasian realms, inhabiting lakes, streams, and puddles, showing a preference for acidic water bodies [15]. At present, molecular data for phylogenetic analysis within the genus *Psectrocladius* remain scarce. Previous research has primarily focused on species delineation and phylogenetic reconstruction through conventional DNA barcoding markers [16]. For instance, morphological characterization of larval and pupal stages in *Psectrocladius nevalis* from Xizang, China, coupled with phylogenetic analysis based on the cytochrome *c* oxidase subunit I (*CO1*) marker, has facilitated the construction of neighbor-joining trees [17]. Recent advancements in methodology have expanded marker selection to include nuclear PCGs, rRNA genes (18S, 28S), and mitochondrial *CO1* for resolving higher-level relationships within Chironomidae [18]. However, phylogenetic studies of *Psectrocladius* remain scarce, particularly regarding the application of complete mitogenome sequences to elucidate evolutionary patterns. As of the latest data in the NCBI Nucleotide database (accessed on 13 April 2025), 283 mitogenomes of Chironomidae have been reported [19,20]. However, no complete mitogenome records are currently available for *Psectrocladius*. This gap underscores the need for mitogenomic approaches to address unresolved phylogenetic uncertainties within the genus.

**Figure 1:**
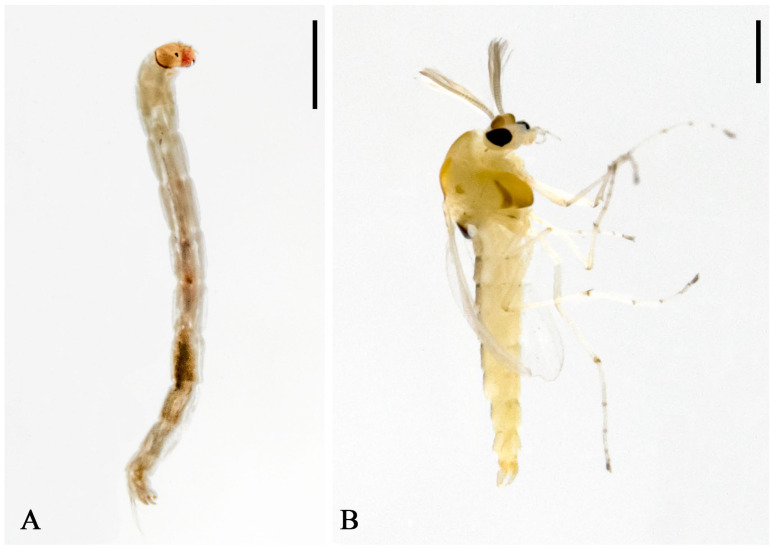
Photos of *Psectrocladius*: (**A**) Larva of *Psectrocladius oligosetus*, Scale bars = 1 mm; (**B**) Adult male of *Psectrocladius schlienzi*, Scale bars = 0.5 mm. Photos were photographed by Xiao-Long Lin, Shanghai Ocean University.

In this study, we provide newly assembled and annotated mitogenomes of five *Psectrocladius* species with annotations (Figure 1). We analyze the basic structural characteristics and subsequently explore the phylogenetic relationships within the genus *Psectrocladius*. Additionally, as the *Psectrocladius* species serve as biological indicators for monitoring the health of freshwater ecosystems, these new mitogenomes provide an essential genomic resource for advancing research on aquatic biodiversity conservation and environmental biomonitoring.

## 2. Materials and Methods

### 2.1. Sampling Collection and DNA Extraction

The specimens of five *Psectrocladius* species analyzed in this study were collected from Xizang, Ningxia, and Sichuan provinces in China between 2014 and 2019. Detailed information is provided in Table 1. The voucher specimens are deposited in the College of Fisheries and Life Science, Shanghai Ocean University, Shanghai, China. Total genomic DNA was extracted from the thoraxes of adults or larvae using the Qiagen DNA blood and tissue Kit (Qiagen, Hilden, Germany). The mitogenomes of the five *Psectrocladius* species were sequenced using the Illumina NovaSeq 6000 platform (Illumina, San Diego, CA, USA) with an insert size of 350 bp and a paired-end 150 bp sequencing strategy at Novogene Co., Ltd. (Beijing, China). The raw reads were trimmed to remove adapters using Trimmomatic [21], and approximately 3 Gb of clean data was obtained for each sample.

### 2.2. Sequencing, Assembly, and Annotation

The seed sequence of CO1 for each species was obtained from GenBank for validation during assembly. The mitogenome sequences were de novo assembled using NOVOPlasty v4.3.1 (Brussels, Belgium) [23] with a k-mer size of 39 and IDBA-UD (Hong Kong, China) [24] with the minimum and maximum k-mer values of 40 and 120 bp, respectively. To verify the accuracy of the mitogenome sequences, we used Geneious v2024.0.5 (Boston, MA, USA) [25] to compare and assemble the obtained sequences into a single contiguous sequence. Transfer RNA (tRNA) genes were detected using MITOS v2.1.7 (Greifswald, Germany) [26]. The rRNAs and PCGs were annotated manually using the Rheocricotopus emeiensis as a reference through Clustal Omega v1.2.4 (Heidelberg, Germany) in Geneious. Finally, the newly assembled mitogenome sequences were submitted to the GenBank database at NCBI (PV132385-PV132389).

### 2.3. Composition Analyses, RSCU, and Evolutionary Rate

Seqkit v2.3.0 (Shenzhen, China) [27] was used to calculate the nucleotide composition and base content bias of the whole mitogenome as well as for each gene category. The AT-skew and GC-skew were computed using the following formulas: AT-skew = [A − T]/[A + T], GC-skew = [G − C]/[G + C]. The relative synonymous codon usage (RSCU) for seven Chironomidae species was determined using MEGA v11 (Philadelphia, PA, USA) [28]. The non-synonymous substitution rate (Ka), synonymous substitution rate (Ks) and their ratios for each PCG were calculated using DnaSP v6.12.01 (Barcelona, Spain) [29] with the ‘mtDNA *Drosophila*’ genetic code. Additionally, nucleotide diversity was evaluated using DnaSP v6.0 (Barcelona, Spain) for DNA polymorphism analysis. Proksee v1.0.1 (Edmonton, AB, Canada) [30], an online platform, was used to generate the visualization of the sequence features of the five mitogenomes.

### 2.4. Phylogenetic Analyses

In this study, five newly sequenced *Psectrocladius* species were selected as in-groups for the phylogenetic analyses, while two species of *Rheocricotopus*, the closest related genus of *Psectrocladius,* were selected as outgroups (Table 1). Phylogenetic analyses were performed separately for the 13 PCGs and the two rRNA genes of each species.

Initially, the 13 PCGs were translated into protein sequences and aligned using MAFFT v7.526 (Kyoto, Japan) [31]. Similarly, the two rRNA genes were aligned based on their nucleotide sequences. Gaps were subsequently removed using trimAl v1.4.1 (Barcelona, Spain) [32], with the parameter “-automated1”. The protein sequences of PCGs were reverse-transcribed into nucleotide sequences for further analysis. After alignment and trimming, the sequences were concatenated and five datasets were generated using FASconCAT-G v1.06.1 (Santa Cruz, CA, USA) [33]: (1) AA dataset, which contains the amino acid sequences translated from 13 PCGs; (2) PCG123 dataset, which contains the nucleotide sequences of all 13 PCGs, including all three codon positions; (3) PCG123_rRNAs dataset, which contains the nucleotide sequences of all 13 PCGs and the two rRNAs; (4) PCG12 dataset, which contains the nucleotide sequences of the 13 PCGs, including only the first and second codon positions; and (5) PCG12_rRNAs dataset, comprising the nucleotide sequences of the 13 PCGs (first and second codon positions) and the two rRNAs. The substitution saturation of all five datasets was measured using DAMBE v7 (Ottawa, ON, Canada) [34], while the heterogeneity analysis was performed by AliGROOVE v.1.08 (Bonn, Germany) [35].

The phylogenetic relationships of the five datasets were inferred with Bayesian inference (BI) and maximum likelihood (ML) methods. BI analysis was conducted by MrBayes v3.2.7 (Stockholm, Sweden) [36], with the best partitioning schemes and the best-fit substitution models for each partition determined by PartitionFinder v2.1.1 (Canberra, Australia) [37]. Markov Chain Monte Carlo (MCMC) was run twice for 10,000,000 generations, with the frequency of tree sampling once every 1000 generations. The initial 25% of the trees from both MCMC runs were discarded as “burn-in”. For the ML analysis, the phylogenetic tree was constructed using IQ-TREE v2.3.6 (Vienna, Austria) [38] with the best-fit substitution model identified and 1000 bootstrap replicates. The resulting trees were visualized and embellished using Figtree v1.4.4 (Edinburgh, UK) [39].

## 3. Results

### 3.1. Mitogenomic Organization

**Figure 2:**
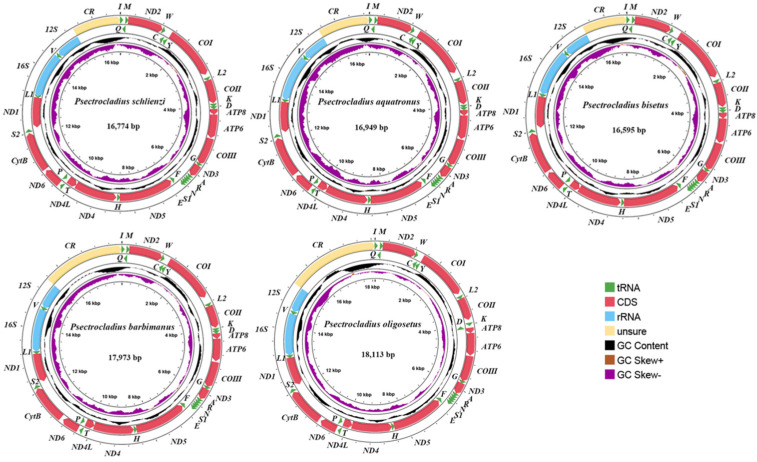
The mitogenome map shows the mitogenomes of five species from *Psectrocladius*. Arrows indicate the direction of gene transcription. Standardized codes are used for PCGs and rRNAs, while single-letter abbreviations were used to denote tRNAs. Different colors represent different types of genes. The second circle highlights the GC content of the entire mitogenome, while the third circle displays the GC-skew. The innermost circle shows the total length of the complete mitogenome.

In this study, we sequenced and assembled the complete mitogenomes of five newly sequenced *Psectrocladius* species (Figure 2). The lengths of these mitogenomes ranged from 16,595 bp (*Psectrocladius bisetus*) to 18,113 bp (*Psectrocladius oligosetus*). The mitogenomes of these species contain 37 typical genes (13 PCGs, two rRNAs, and 22 tRNAs) and one CR, a structure commonly found in most insect mitogenomes. The lengths of the 13 PCGs, tRNAs, l-rRNA, s-rRNA changed slightly, ranging from 11,211 bp (*Psectrocladius aquatronus*) to 11,217 bp (*Psectrocladius barbimanus*), 1485 bp (*P. bisetus*) to 1493 bp (*P. aquatronus*), 1398 bp (*P. oligosetus*) to 1438 bp (*P. aquatronus*) and 802 bp (*P. bisetus* and *P. oligosetus*) to 807 bp (*P. barbimanus*), respectively. In contrast, the length of CR varied significantly, ranging from 1212 bp (*P. bisetus*) to 2719 bp (*P. oligosetus*).

### 3.2. Nucleotide Composition

The nucleotide composition of the five newly obtained mitogenomes was consistent with typical insect mitogenomes, exhibiting a pronounced A + T bias, ranging from 73.59% in *P. oligosetus* to 76.56% in *P. barbimanus*. The control region (CR) showed the highest A + T content, ranging from 79.62% (*P. aquatronus*) to 87.24% (*Psectrocladius schlienzi*), whereas the PCGs exhibited the lowest A + T content (70.34% to 72.87%). Within the PCGs, the A + T bias was lower in the first and second codon positions compared to the third codon position, which exhibited the highest A + T content. In tRNAs, the A + T content was lower than that in rRNAs.

The analysis revealed a positive AT-skew across all mitogenomes, whereas the GC-skew was negative. Specifically, the AT-skew was positive in the tRNA and CR regions but negative in the two rRNAs and all three codon positions within the PCGs. The GC-skew was negative in the PCGs and CR but positive in rRNAs, tRNA and the first codon position of the PCGs. The detailed results of the nucleotide composition and skew analysis are presented in Table S1.

### 3.3. Codon Usage of PCGs

**Figure 3:**
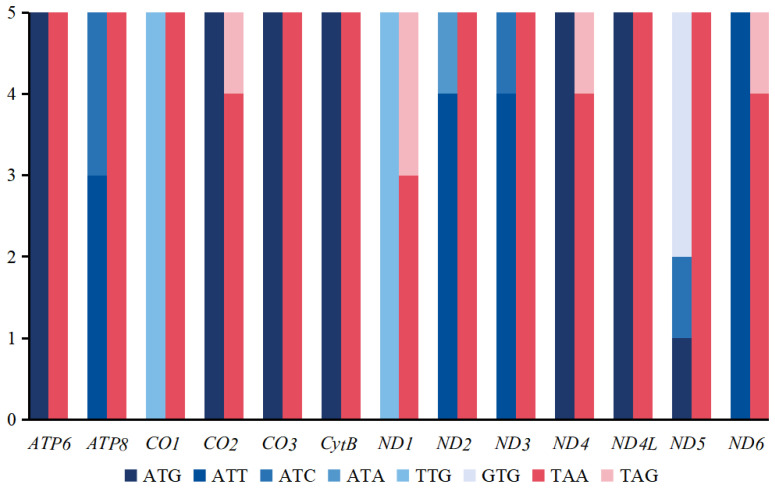
Start and stop codons of PCGs among five species of *Psectrocladius*. The *x*-axis represents the 13 PCGs, while the *y*-axis shows the number of different codons. The varying shades of blue indicate the start codons, and the two shades of red represent the stop codons.

**Figure 4:**
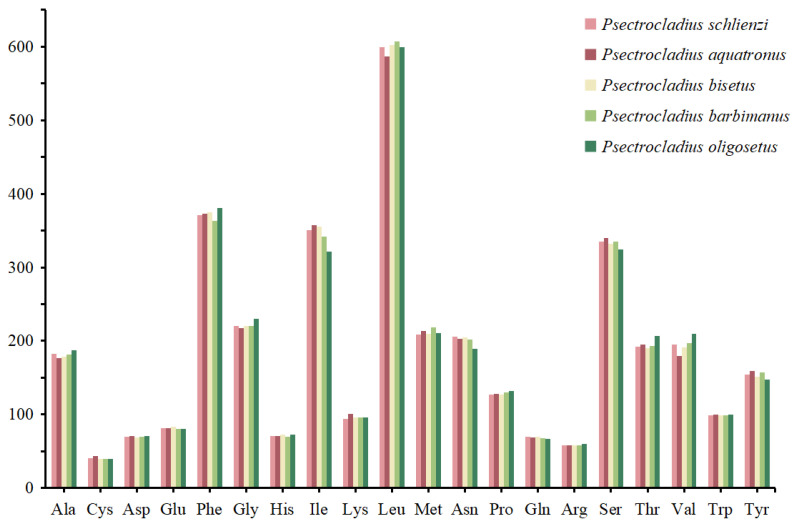
Pattern of codon usage of mitogenomes from five species of *Psectrocladius*. The *x*-axis represents the codon families, while the *y*-axis shows the total codons.

The distribution of start and stop codons in the 13 PCGs is shown in Figure 3 and Table S1. Most genes exhibited the typical ATN start codon pattern, except for *CO1* and *ND1* genes, which both started with the TTG codon. Additionally, in the *ND5* gene, the start codon was GTG in three species. Regarding stop codons, all five species consistently used the typical TAA or TAG patterns for termination. The frequency of codon families was similar among the five *Psectrocladius* species (Figure 4). The most common codon families were Leu, Phe and Ile, while the least frequent codon family was Cys, consistent with findings from other studies on chironomids [40,41].

### 3.4. Substitution Rates and Nucleotide Diversity

**Figure 5:**
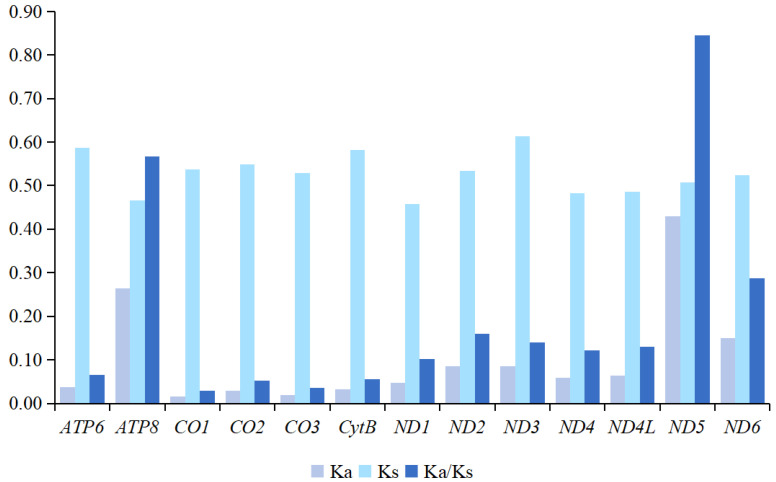
The evolutionary rates of the 13 PCGs in species from *Psectrocladius*. Ka refers to non-synonymous nucleotide substitutions, Ks refers to synonymous nucleotide substitutions, and Ka/Ks refers to the selection pressure acting on each PCG. The *x*-axis represents the 13 PCGs, while the *y*-axis shows the Ka/Ks values. The varying shades of blue represent the values for Ka, Ks and Ka/Ks.

As shown in Figure 5 and Table S3, the ratio of Ka/Ks ranged from 0.03 (*CO1*) to 0.85 (*ND5*), with all 13 PCGs showing values below 1, indicating that all these genes are under negative selection pressure. The strongest purifying selection was observed in the *CO1* gene, while the weakest was in the *ND5* gene. In terms of non-synonymous substitution rates, *ND5* exhibited the highest Ka value (0.43), whereas *CO1* and *CO3* had the lowest (0.02). The highest substitution rate was observed in *ND3* (0.61), while *ND1* displayed the lowest (0.46). The nucleotide diversity (Pi) varied significantly across the 13 PCGs. The highest Pi was found in the *ND5* gene (0.45), while *CO3* had the lowest Pi value, with 0.13.

### 3.5. Heterogeneity Analyses of Mitogenomes

**Figure 6:**
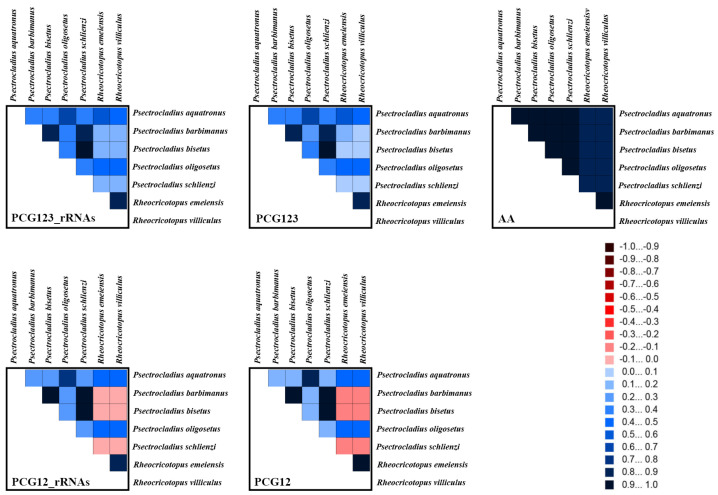
The heterogeneity of the mitogenomes of seven species from *Psectrocladius* and *Rheocricotopus* based on PCGs, rRNAs and amino acids. Sequence similarity is visualized using colored blocks, with AliGROOVE scores ranging from −1 (indicating strong heterogeneity between datasets, represented by red) to +1 (indicating weak heterogeneity, represented by blue). Lighter-colored blocks correspond to higher heterogeneity, while darker-colored blocks indicate lower heterogeneity.

The five datasets analyzed in this study contained the following site counts: (1) AA dataset contained 3723 sites; (2) PCG123 dataset contained 11,169 sites; (3) PCG123_rRNAs dataset contained 13,329 sites; (4) PCG12 dataset contained 7446 sites; and (5) PCG12_rRNAs dataset contained 9606 sites. Heterogeneity reflects the degree of similarity of mitogenome sequences among different species. The results of the heterogeneity analysis are shown in Figure 6. The AA dataset exhibited the lowest random similarity, indicating that even when changes occur at the third codon position, these tend to transition to synonymous codons that encode the same amino acid [41].

Generally, the third codon position of PCGs evolves faster than the first and second positions. However, our results indicate that the heterogeneity of the PCG123 dataset was lower than that of the PCG12 dataset, while the heterogeneity of the PCG12_rRNAs dataset was higher than that of the PCG123_rRNAs dataset. This discrepancy may be attributed to strong selective constraints acting on the third codon position in the mitogenome, which plays a crucial functional role. Although the mutation rate at the third codon position is higher, the types and frequencies of mutations may be constrained by purifying selection, thereby reducing heterogeneity. Alternatively, certain genes may have reached mutation saturation at the third codon position, resulting in lower variability, whereas the first and second codon positions, which have a greater impact on amino acid changes, may exhibit lower variability due to stronger selective pressure.

### 3.6. Phylogenetic Analysis

**Figure 7:**
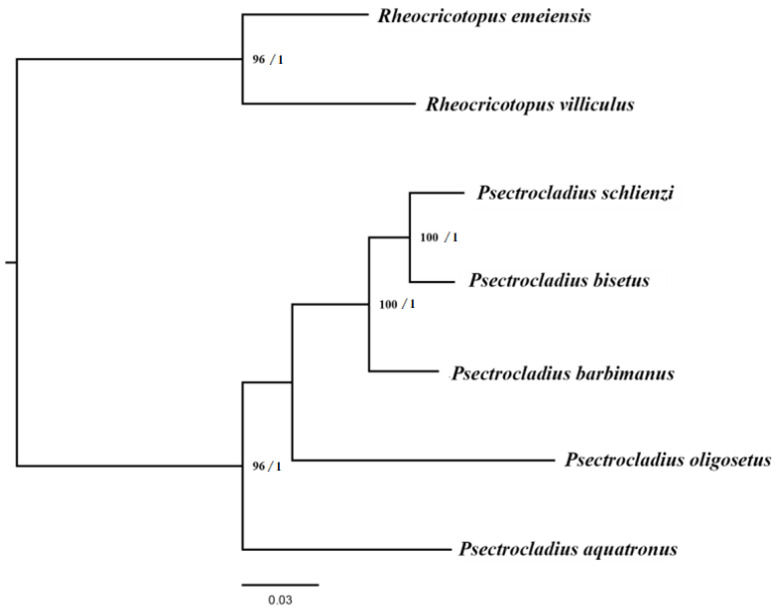
Phylogenetic tree of *Psectrocladius* inferred from the AA dataset based on the ML and BI methods. Nodes are labeled as support values from both analyses, including bootstrap support (BS), is shown to the left of the slash and Bayesian posterior probabilities (PP) to the right. Nodes with BS < 70 and PP < 0.95 are considered to have weak support and are not annotated.

The results for each dataset revealed that the *ISS* value was lower than *ISS*. *c*, with *p* < 0.05, indicating that substitutions had not reached saturation and that the datasets were suitable for constructing the phylogenetic relationships. The results of DAMBE are shown in Figure S1. The best-fit substitution models for each partition of the five matrices are shown in Table S2. The topologies of the phylogenetic trees were consistent across all datasets, with the overall shapes of the trees being approximately the same (Figure 7 and Figure S2–S5). Specifically, the monophyly of *Psectrocladius* was well supported, with all five *Psectrocladius* species grouped into a single clade. Within *Psectrocladius*, the phylogeny showed the following relationships: ((((*P. schlienzi* + *P. bisetus*) + *P. barbimanus*) + *P. oligosetus*) + *P. aquatronus*).

## 4. Conclusions

The present study provides the first complete mitogenomes of five *Psectrocladius* species, along with an analysis of their general features and phylogenetic relationships within the genus. Comparative analyses reveal that the mitogenomes of *Psectrocladius* are structurally conserved and exhibit a putative ancestral gene arrangement. The nucleotide composition of the five mitogenomes exhibits distinct A + T bias. Most PCGs initiate with the ATN start codon pattern, except for *CO1*, *ND1* and *ND5* genes, which use TTG and GTG as start codons. Evolutionary rates vary significantly among species, and the length and nucleotide composition of the CR also exhibit high variability. *CO1* and *ND1* may serve as effective molecular markers for the classification of the *Psectrocladius* species. Phylogenetic analyses based on mitogenomes support the monophyly of *Psectrocladius*. Both the ML and Bayesian trees consistently reveal the following topology: ((((*P. schlienzi* + *P. bisetus*) + *P. barbimanus*) + *P. oligosetus*) + *P. aquatronus*).

## Figures and Tables

**Table 1:** Detailed information of five *Psectrocladius* and two *Rheocricotopus* species used in the study.

Species	Sample ID	Life Stage	Sampling Metadata	GenBank Accession	Reference
*Psectrocladius schlienzi*	NAM65	Adult male	Xigaze, Xizang, China, 29.317° N, 88.934° E, 22 July 2014, leg. X.-L. Lin	PV132385	this study
*Psectrocladius barbimanus*	XL4313	Adult male	Nagqu, Xizang, China, 31.733° N, 87.899° E, 6 September 2019, leg. E.-L. Zhang	PV132387	this study
*Psectrocladius aquatronus*	NX001	Adult male	Wuzhong, Ningxia, China, 37.737° N, 107.352° E, 27 April 2018, leg. L. Kang	PV132386	this study
*Psectrocladius bisetus*	NX009	Adult male	Wuzhong, Ningxia, China, 37.87° N, 107.57° E, 7 May 2018, leg. L. Kang	PV132389	this study
*Psectrocladius oligosetus*	XL3277	Larva	Jiuzhaigou, Sichuan, China, 33.228° N, 103.897° E, 12 July 2019, leg. X.-Y. Ge	PV132388	this study
*Rheocricotopus emeiensis*	XL1426	Adult male	Leshan, Sichuan, China, 29.574° N, 103.356° E, 23 July 2017, leg. C. Song	OP006246	[13]
*Rheocricotopus villiculus*	ZJ497	Adult male	Hangzhou, Zhejiang, China, 30.322° N, 119.442° E, 22 July 2019, leg. X.-L. Lin	MW373526	[22]

## Data Availability

The following information was supplied regarding the availability of DNA sequences: the new mitogenomes are deposited in GenBank of NCBI under the accession numbers PV132385–PV132389.

## Supplementary Materials

The following supporting information can be downloaded at: https://www.mdpi.com/article/10.3390/insects16040420/s1, Table S1: Nucleotide composition of mitogenomes of five *Psectrocladius* species; Table S2: The best model for each partition of the five datasets; Table S3: Nucleotide diversity (Pi) values of seven species of PCGs; Figure S1: Substitution patterns of the PCG12 (a), PCG12_rRNAs (b), PCG123 (c) and PCG123_rRNAs (d) datasets. The graphs represent the increase in GTR distance; Figure S2: Phylogenetic trees of *Psectrocladius* inferred from the PCG12 dataset. (a) BI tree. Numbers at the nodes are BI posterior probabilities. (b) ML tree. Numbers at the nodes are ML bootstrap values; Figure S3: Phylogenetic trees of *Psectrocladius* inferred from the PCG12_rRNAs dataset. (a) BI tree. Numbers at the nodes are BI posterior probabilities. (b) ML tree. Numbers at the nodes are ML bootstrap values; Figure S4: Phylogenetic trees of *Psectrocladius* inferred from the PCG123 dataset. (a) BI tree. Numbers at the nodes are BI posterior probabilities. (b) ML tree. Numbers at the nodes are ML bootstrap values; Figure S5: Phylogenetic trees of *Psectrocladius* inferred from the PCG123_rRNAs dataset. (a) BI tree. Numbers at the nodes are BI posterior probabilities. (b) ML tree. Numbers at the nodes are ML bootstrap values.
