# Supplementary material for: Comparative Mitogenomic Analyses of Psectrocladius (Diptera: Chironomidae)
Source: Insects. 2025 Apr 16;16(4):420. doi: 10.3390/insects16040420 (PMC12027813; doi:10.3390/insects16040420)
Supplement: Supplementary file 1 [file insects-16-00420-s001.zip › insects-3586084-supplementary.pdf]

Supplementary materials

Table S1. Nucleotide composition of mitogenomes of five *Psectrocladius* species.

Regions	Species	mitogenome	PCGs	1st Codon Position	2nd Codon Position	3rd Codon Position	tRNA	16SrRNA	12SrRNA	CR
Length (bp)	*Psectrocladius schlienzi*	1,7674	11,214	3,738	3,738	3,738	1,490	1,422	803	1,364
	Psectrocladius aquatronus	16,949	11,211	3,737	3,737	3,737	1,493	1,438	806	1,457
	Psectrocladius bisetus	16,595	11,214	3,738	3,738	3,738	1,485	1,405	802	1,212
	Psectrocladius barbimanus	17,973	11,217	3,739	3,739	3,739	1,492	1,417	807	2,439
	Psectrocladius oligosetus	18,113	11,214	3,738	3,738	3,738	1,492	1,398	802	2,719
A+T%	*Psectrocladius schlienzi*	75.18	71.46	67.12	66.45	80.79	78.52	82.35	81.32	87.24
	Psectrocladius aquatronus	75.64	72.67	68.02	66.34	83.65	78.97	83.66	81.64	79.62
	Psectrocladius bisetus	75.26	71.86	67.6	66.69	81.27	78.52	82.42	81.67	87.21
	Psectrocladius barbimanus	76.56	72.87	67.8	66.41	84.41	79.02	83.56	81.29	82.53
	Psectrocladius oligosetus	73.59	70.34	65.7	65.76	79.56	76.54	81.47	79.93	79.74
G+C%	*Psectrocladius schlienzi*	24.82	28.54	32.88	33.55	19.21	21.48	17.65	18.68	12.76
	Psectrocladius aquatronus	24.36	27.33	31.98	33.66	16.35	21.03	16.34	18.36	20.38
	Psectrocladius bisetus	24.74	28.14	32.4	33.31	18.73	21.48	17.58	18.33	12.79
	Psectrocladius barbimanus	23.44	27.13	32.2	33.59	15.59	20.98	16.44	18.71	17.47
	Psectrocladius oligosetus	26.41	29.66	34.3	34.24	20.44	23.46	18.53	20.07	20.26

AT-Skew	*Psectrocladius schlienzi*	0.05	-0.17	-0.08	-0.39	-0.08	0.03	-0.10	-0.01	0.10
	Psectrocladius aquatronus	0.05	-0.17	-0.07	-0.38	-0.08	0.01	-0.09	-0.04	0.09
	Psectrocladius bisetus	0.05	-0.18	-0.08	-0.39	-0.08	0.02	-0.13	-0.02	0.07
	Psectrocladius barbimanus	0.06	-0.17	-0.08	-0.39	-0.08	0.03	-0.12	-0.02	0.12
	Psectrocladius oligosetus	0.05	-0.18	-0.08	-0.40	-0.09	0.02	-0.08	-0.01	0.08
GC-Skew	*Psectrocladius schlienzi*	-0.18	-0.02	0.22	-0.17	-0.16	0.17	0.29	0.28	-0.23
	Psectrocladius aquatronus	-0.19	-0.02	0.22	-0.15	-0.23	0.17	0.32	0.27	-0.29
	Psectrocladius bisetus	-0.18	-0.02	0.23	-0.16	-0.21	0.16	0.34	0.28	-0.29
	Psectrocladius barbimanus	-0.17	0.01	0.24	-0.16	-0.13	0.17	0.30	0.27	-0.26
	Psectrocladius oligosetus	-0.17	-0.01	0.21	-0.16	-0.14	0.13	0.34	0.30	-0.20

Table S2. The best model for each partition of the five datasets.

Datasets	Partition names	Best model
PCG12	CO1_pos1, CO3_pos1, ATP6_pos1, CytB_pos1, CO2_pos1	GTR+G
PCG12	CO2_pos2, CytB_pos2, CO3_pos2, ATP6_pos2, CO1_pos2	GTR+I+G
PCG12	ND3_pos1, ND6_pos1, ATP8_pos2, ATP8_pos1	GTR+I+G
PCG12	ND1_pos1, ND4_pos1, ND4L_pos1	GTR+I+G
PCG12	ND6_pos2, ND3_pos2, ND1_pos2, ND4L_pos2, ND4_pos2	GTR+I+G
PCG12	ND2_pos1, ND5_pos1	GTR
PCG12	ND2_pos2, ND5_pos2	GTR
PCG12R	CO3_pos1, CO1_pos1, ATP6_pos1, CO2_pos1, CytB_pos1	GTR+G
PCG12R	CO1_pos2, CO3_pos2, ATP6_pos2, CO2_pos2, CytB_pos2	GTR+I+G
PCG12R	ATP8_pos1, ATP8_pos2, ND3_pos1, ND6_pos1	GTR+I+G
PCG12R	ND1_pos1, ND4_pos1, ND4L_pos1	GTR+I+G
PCG12R	ND6_pos2, ND1_pos2, ND3_pos2, ND4L_pos2, ND4_pos2	GTR+G
PCG12R	ND2_pos1, ND5_pos1	GTR
PCG12R	ND2_pos2, ND5_pos2	GTR
PCG12R	12S_gene, 16S_gene	GTR+I+G
PCG123	CO3_pos1, CO1_pos1, ATP6_pos1, CytB_pos1, CO2_pos1	GTR+I+G
PCG123	CO1_pos2, CO3_pos2, ND3_pos2, ATP6_pos2, CytB_pos2, CO2_pos2	GTR+I+G
PCG123	ND3_pos3, ATP6_pos3, CO1_pos3, CO2_pos3, CO3_pos3, CytB_pos3	GTR+I+G
PCG123	ATP8_pos2, ATP8_pos1, ND3_pos1, ND6_pos1	GTR+I+G
PCG123	ND2_pos3, ND6_pos3, ATP8_pos3	GTR+G
PCG123	ND1_pos1, ND4_pos1, ND4L_pos1	GTR+I+G
PCG123	ND6_pos2, ND4L_pos2, ND4_pos2, ND1_pos2	GTR+G
PCG123	ND1_pos3, ND4L_pos3, ND4_pos3	GTR+I+G
PCG123	ND2_pos1, ND5_pos1	GTR
PCG123	ND5_pos2, ND2_pos2	GTR
PCG123	ND5_pos3	GTR
PCG123R	CO3_pos1, CO1_pos1, ATP6_pos1, CytB_pos1, CO2_pos1	GTR+I+G
PCG123R	CO1_pos2, CO3_pos2, ND3_pos2, ATP6_pos2, CytB_pos2, CO2_pos2	GTR+I+G
PCG123R	ND3_pos3, ATP6_pos3, CO1_pos3, CO2_pos3, CO3_pos3, CytB_pos3	GTR+I+G
PCG123R	ATP8_pos1, ATP8_pos2, ND3_pos1, ND6_pos1	GTR+I+G
PCG123R	ND2_pos3, ATP8_pos3, ND6_pos3	GTR+G
PCG123R	ND1_pos1, ND4_pos1, ND4L_pos1	GTR+I+G
PCG123R	ND6_pos2, ND4L_pos2, ND4_pos2, ND1_pos2	GTR+G
PCG123R	ND1_pos3, ND4L_pos3, ND4_pos3	GTR+I+G
PCG123R	ND2_pos1, ND5_pos1	GTR

	ND5_pos2, ND2_pos2	GTR
	ND5_pos3	GTR
	16S_gene, 12S_gene	GTR+G
AA	CytB, ATP6, CO2	MTMAM+G
	ATP8, ND6	MTREV+G
	CO3, CO1	MTMAM+G
	ND5, ND1, ND3, ND4, ND4L	MTMAM+I+G
	ND2	MTREV+G

Table S3. Nucleotide diversity (Pi) values of five species of PCGs.

Gene	Pi
ATP6	0.17
ATP8	0.30
CO1	0.14
CO2	0.14
CO3	0.13
CytB	0.16
ND1	0.14
ND2	0.18
ND3	0.19
ND4	0.15
ND4L	0.15
ND5	0.45
ND6	0.23

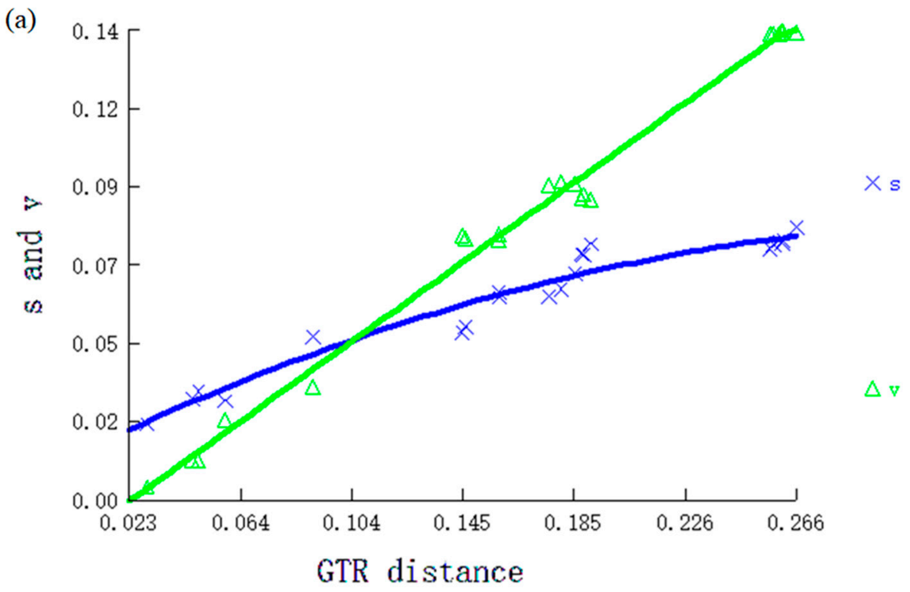

(a)
0. 14
0. 12
0. 09
0. 07
0. 05
0. 02
0. 00
s and v
0. 023
0. 064
0. 104
0. 145
0. 185
0. 226
0. 266
GTR distance
s
v

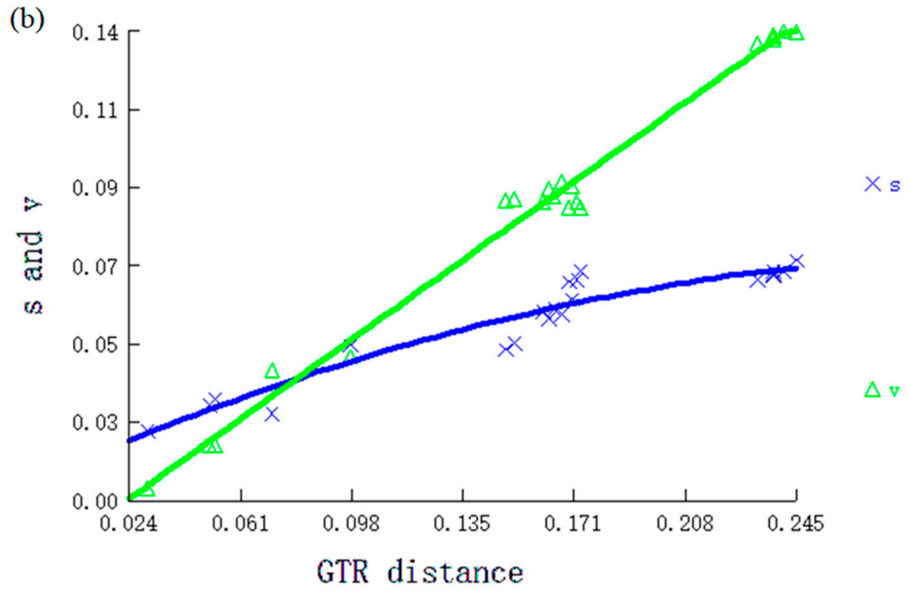

(b)
0. 14
0. 11
0. 09
0. 07
0. 05
0. 03
0. 00
s and v
0. 024
0. 061
0. 098
0. 135
0. 171
0. 208
0. 245
GTR distance
s
v

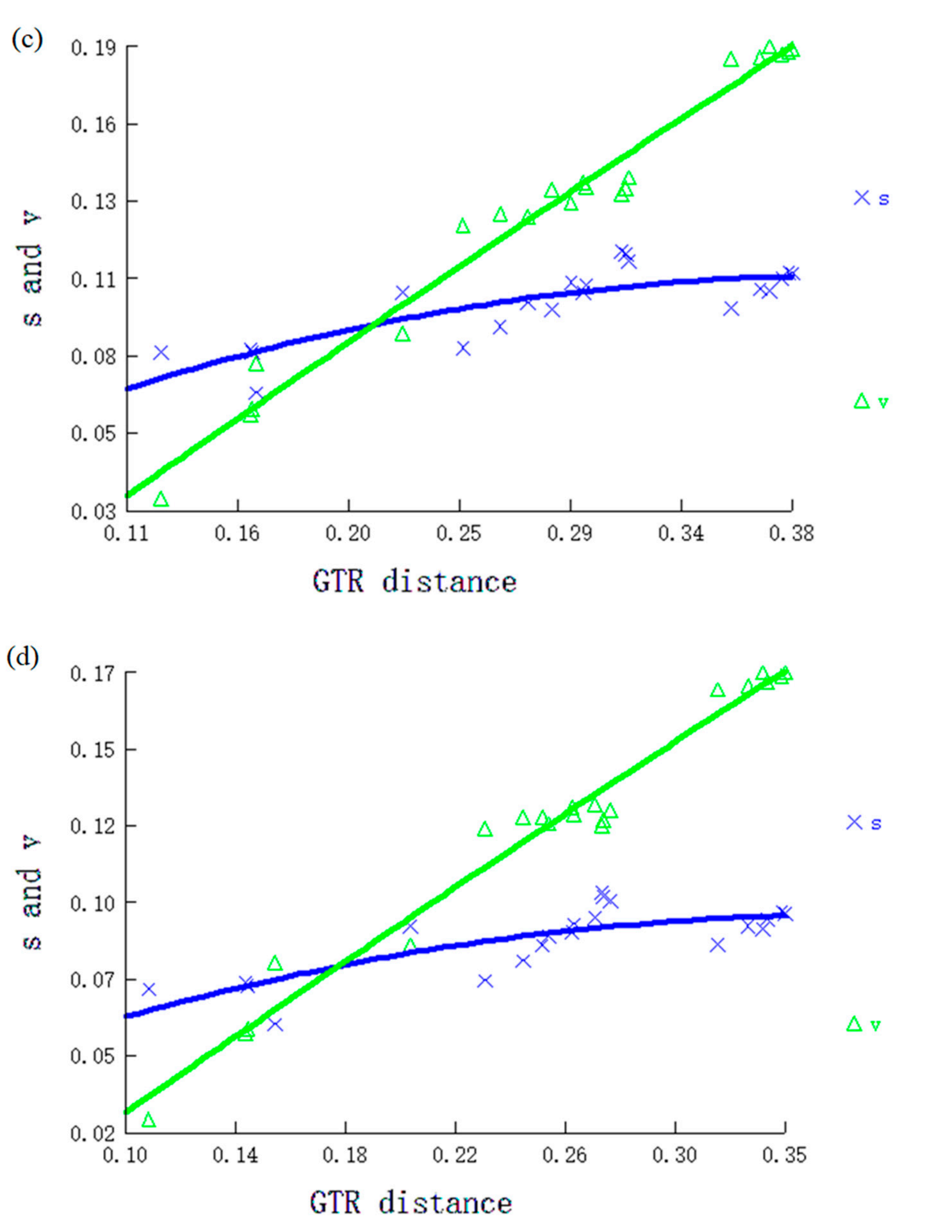

Figure S1. Substitution patterns of the PCG12 (a), PCG12R (b), PCG123 (c) and PCG123R (d) datasets. The graphs represent the increase in GTR distance.

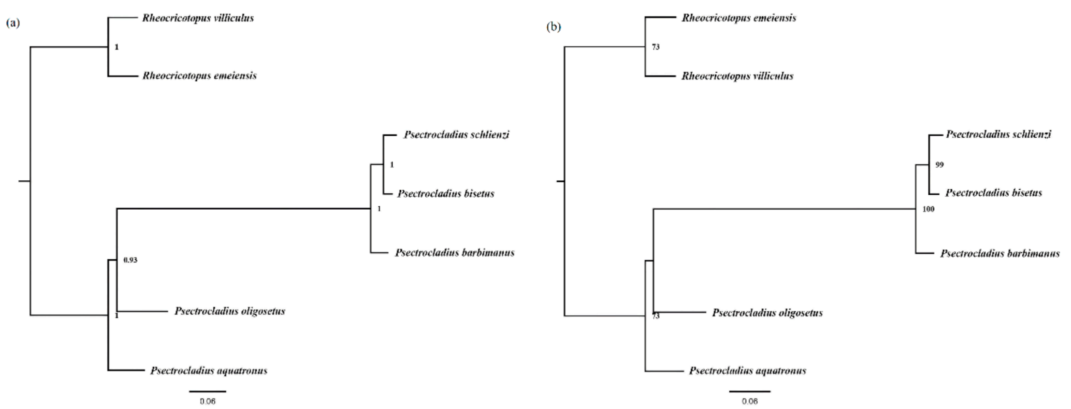

Figure S2. Phylogenetic trees of *Psectrocladius* inferred from the PCG12 dataset. (a) BI tree. Numbers at the nodes are BI posterior probabilities. (b) ML tree. Numbers at the nodes are ML bootstrap values.

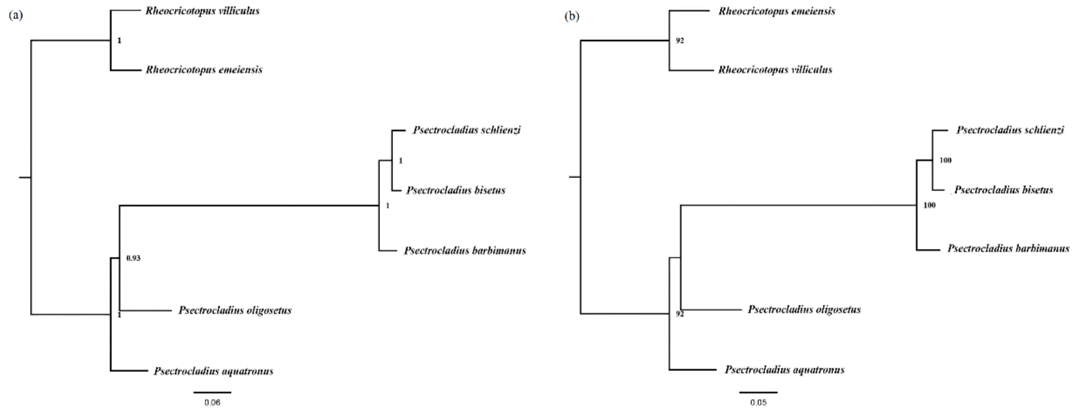

Figure S3. Phylogenetic trees of *Psectrocladius* inferred from the PCG12R dataset. (a) BI tree. Numbers at the nodes are BI posterior probabilities. (b) ML tree. Numbers at the nodes are ML bootstrap values.

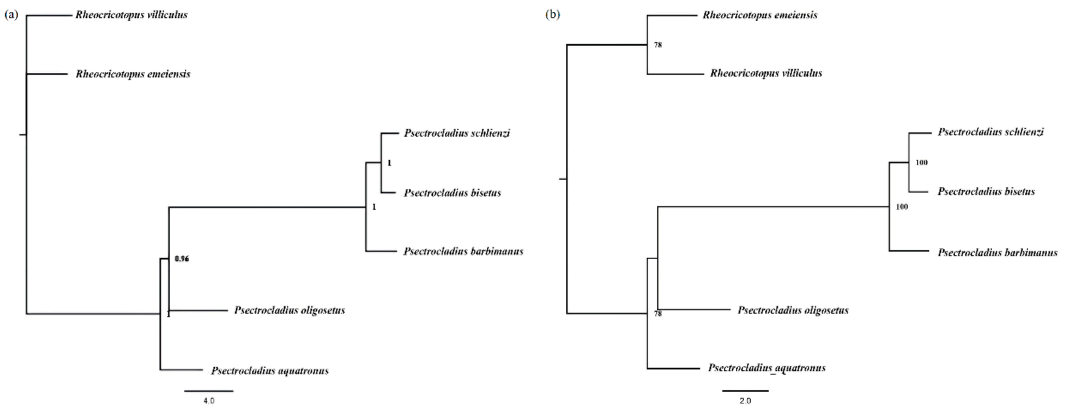

Figure S4. Phylogenetic trees of *Psectrocladius* inferred from the PCG123 dataset. (a) BI tree. Numbers at the nodes are BI posterior probabilities. (b) ML tree. Numbers at the nodes are ML bootstrap values.

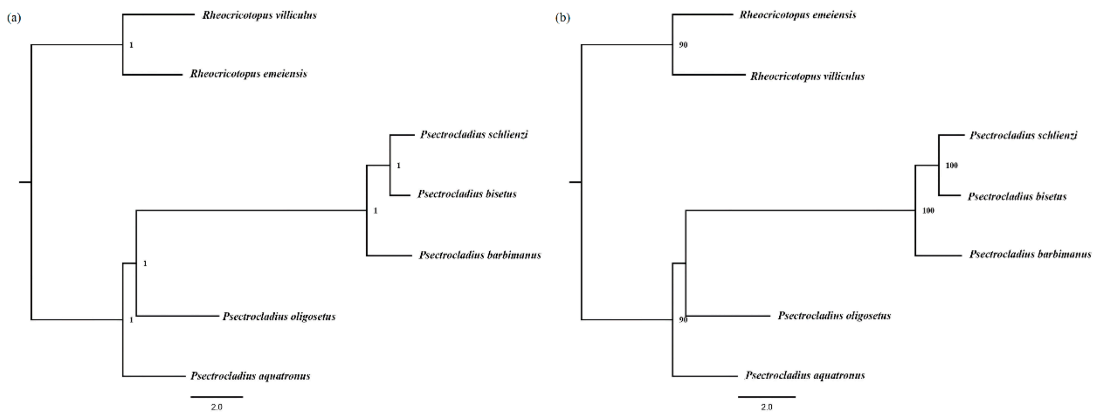

Figure S5. Phylogenetic trees of *Psectrocladius* inferred from the PCG123R dataset. (a) BI tree. Numbers at the nodes are BI posterior probabilities. (b) ML tree. Numbers at the nodes are ML bootstrap values.
